# Malaria among foreign migrant workers in Savannakhet Province, Lao People’s Democratic Republic

**DOI:** 10.1186/s41182-019-0140-2

**Published:** 2019-01-25

**Authors:** Tiengkham Pongvongsa, Daisuke Nonaka, Moritoshi Iwagami, Pheovaly Soundala, Phonepadith Khattignavong, Phonepadith Xangsayarath, Futoshi Nishimoto, Jun Kobayashi, Bouasy Hongvanthon, Paul T. Brey, Shigeyuki Kano

**Affiliations:** 1 Savannakhet Provincial Health Department, Phonsavangnuea village, Kaysone-Phomvihan district, Savannakhet, Lao PDR; 2 SATREPS Project for Parasitic Diseases, Vientiane Capital, Lao PDR; 3 Department of Global Health, School of Health Sciences, University of the Ryukyus, 207 Uehara, Nishihara-cho, Okinawa, 903-0215 Japan; 4 Department of Tropical Medicine and Malaria, Research Institute, National Center for Global Health and Medicine, 1-21-1 Toyama, Shinjuku-ku, Tokyo, 162-8655 Japan; 5 Institut Pasteur du Laos, Ministry of Health, Sisattanak district, Vientiane Capital, Lao PDR; 6 National Center for Laboratory and Epidemiology, Ministry of Health, Sisattanak district, Vientiane Capital, Lao PDR; 7 Graduate School of International Health Development, Nagasaki University, 1-12-4 Sakamoto, Nagasaki-shi, Nagasaki, 852-8523 Japan; 8 Center of Malariology, Parasitology and Entomology, Ministry of Health, Sisattanak district, Vientiane Capital, Lao PDR

**Keywords:** Malaria, Labor migration, International migration, Laos, Vietnam, Risk factor, Help-seeking behavior

## Abstract

**Background:**

Although mobile and migrant populations are considered an important group in malaria elimination settings, there is currently a lack of understanding about foreign migrant workers in the Lao People’s Democratic Republic (Lao PDR). The present study aimed to document the migration characteristics, positive rate of malaria infection, and preventive and treatment-seeking behavior for malaria among foreign migrant workers in the malaria-endemic districts of Savannakhet province, Lao PDR.

**Methods:**

A community-based survey was undertaken in four districts of Savannakhet province between February and June, 2015. Questionnaire-based interviews and blood examinations, including rapid diagnostic tests and PCR assays, were conducted with 391 migrant workers who were registered at local police departments.

**Results:**

Most of the study participants were men (75.7%) and Vietnamese (92.6%). The median age (interquartile range) was 31 (25 to 41) years old. Most common occupation was factory worker (47.6%), followed by trader/shopkeeper (21.5%) and plantation worker/farmer (16.4%). The median length of stay (interquartile range) in the districts was 405 (183 to 1207) days. The majority of the participants (85.9%) had not worked in a province other than the study province, nor had the majority (92.6%) worked in a foreign country other than the Lao PDR. Although most of the participants (62.7%) reportedly used a bed net daily, these nets were mostly conventional untreated ones. No one tested positive for malaria. However, 10.0% of the participants reported a malaria-like illness episode that had occurred in the Lao PDR. The most common measure taken for the episode was to visit a hospital/health center in the Lao PDR, followed by conducting self-medication alone. Forty-one participants reported an experience of working in the forest while living in the Lao PDR.

**Conclusions:**

Foreign migrant workers who are registered at local police departments are unlikely to play a major role in maintaining local transmissions and spreading drug-resistant malaria in the study province. However, some of them were involved in forest-related activities, suggesting that these workers are potentially at risk of malaria. The Lao National Malaria Control Program should educate foreign migrant workers about the risk of malaria when living in Lao PDR.

## Background

The burdens of malaria have decreased in the Lao People’s Democratic Republic (Lao PDR) in recent years: estimated malaria cases dropped from 51,000 in 2010 to 27,390 in 2016 [1]. A current challenge to malaria control in the Lao PDR is the emergence of drug-resistant malaria [2]. In 2015, the countries in the Greater Mekong Sub-region (GMS) such as Cambodia, China (specifically Yunnan Province and Guangxi Zhuang Autonomous Region), Lao PDR, Myanmar, Thailand, and Vietnam adopted the malaria elimination strategy that was proposed by the World Health Organization because *Plasmodium falciparum* resistance to antimalarial medicines had reached alarming levels in certain areas of the GMS, and the only solution is to eliminate *P. falciparum* from the GMS. The GMS countries have been working to eliminate all species of human malaria by 2030 [3].

The Asian Highway and GMS economic corridors that cut through endemic areas in GMS countries including Lao PDR are contributing to the increased population movement between and within GMS countries [4]. In response to the recent rapid economic development in the Lao PDR, increasing numbers of migrant workers are entering the Lao PDR from Vietnam, China, Thailand, and Myanmar. For example, hydropower dam projects in Attapeau province involved an estimated 4000 to 5000 workers at the peak of the construction phase in 2012, the majority of whom were Vietnamese and Chinese [5]. There are estimated to be 54,000 foreign migrant workers in the Lao PDR in 2017 [6].

The National Malaria Control Program of the Lao PDR has paid special attention to migrant workers for a number of reasons. First, migrant workers are likely to be involved in spreading drug-resistant malaria from one area to another. Second, migrant workers are considered to be at high risk of contracting malaria. In a large outbreak that occurred in Attapeau province, migrants, both from other provinces in the Lao PDR and from neighboring countries, accounted for the majority of the reported cases [2, 5]. Finally, foreign migrants are particularly likely to contribute to maintaining local transmissions as they may be less likely to seek treatment from a local healthcare service compared to those of Lao nationality [5, 7].

**Fig. 1:**
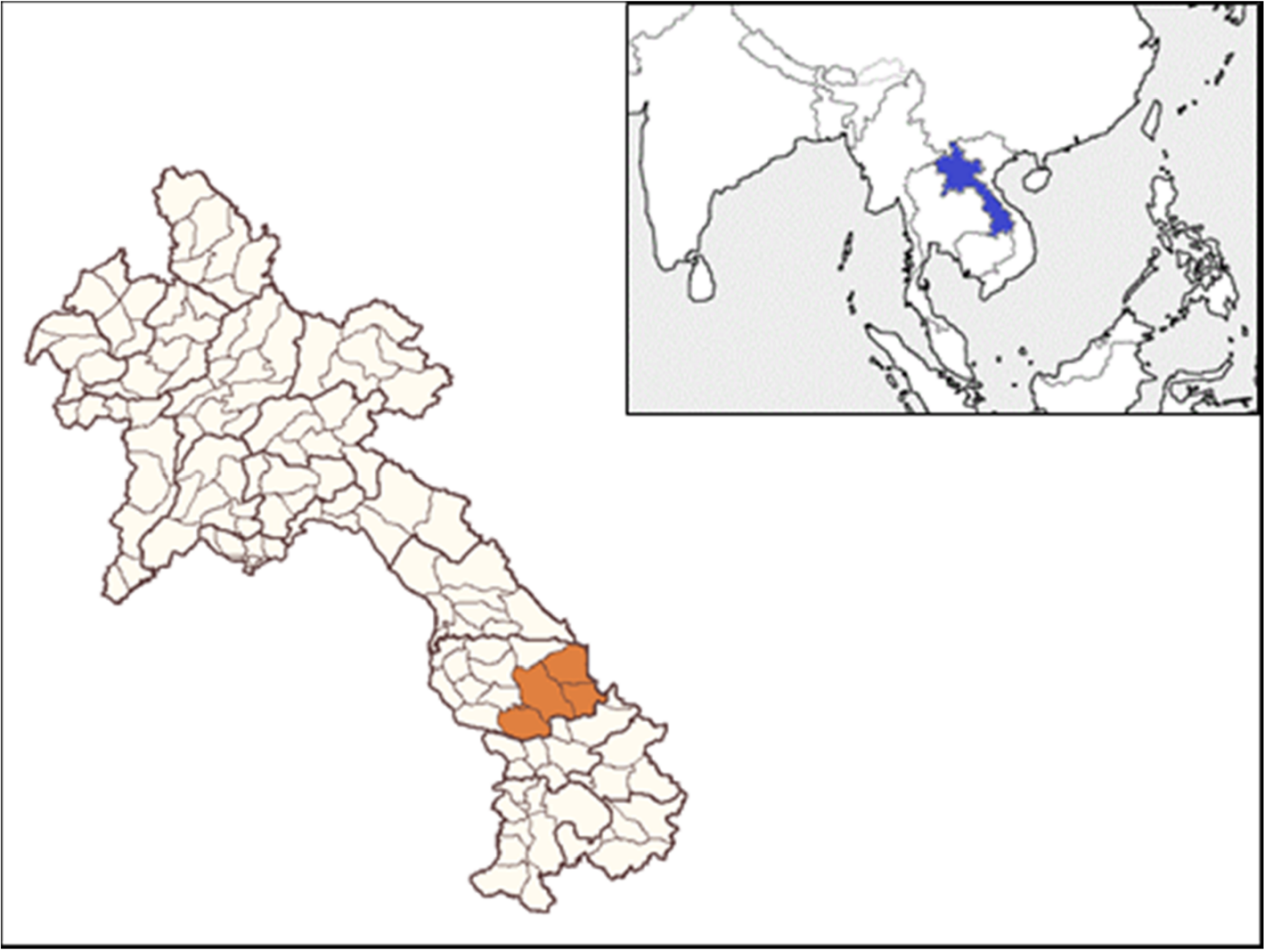
Map of the Lao PDR and the study districts. The small map insert at the upper right shows the location of the Lao PDR highlighted in blue. The large map shows the location of the four study districts of Thapangthong, Nong, Xepon, and Phin highlighted in brown

Among the 18 provinces of the Lao PDR, Savannakhet province is a major province to which Vietnamese emigrate [8]. As the largest province (21,774 km^2^) in the Lao PDR, it is bordered by Vietnam to the east and Thailand to the west. Approximately 90% of all malaria cases in the Lao PDR occur in the five southernmost provinces, of which Savannakhet is one [9]. Of the 15 districts in Savannakhet province, malaria is endemic in four districts: Thapangthong, Nong, Xepon, and Phin (Fig. 1).

There is currently a lack of understanding about foreign migrant workers in the Lao PDR in terms of designing and implementing malaria control strategies to better target them. Therefore, the present study aimed to document the migration characteristics, positive rate of malaria infection, and preventive and treatment-seeking behavior for malaria among foreign migrant workers in the four malaria-endemic districts of Savannakhet province, Lao PDR.

## Methods

### Study site and participants

The present study was conducted in the four districts of Thapangthong, Nong, Xepon, and Phin in Savannakhet province, whose populations in 2015 were 40,584, 29,622, 56,213, and 65,085, respectively [10]. Prior to field survey, the research team estimated the number of registered foreign migrant workers to be around 100 to 150 in each district, on the basis of the information from relevant authorities.

In each district, the research team visited the local police office, where local residents are registered, to collect information on the residences and workplaces of foreign migrant workers. Then, the team visited the residences and/or workplaces of these workers with the help of the local authorities, including village heads. When an eligible foreign migrant worker was absent, multiple attempts were made to contact him/her.

The research team included in the present study all of the foreign migrant workers on whom information was provided by the local police office. Foreign migrant workers were excluded if they were aged less than 15 years old or refused to participate in the study. Out of a total of 520 registered foreign migrant workers (Thapangthong 100, Xepon 150, Nong 120, and Phin 150), 392 participated in the study (participation rate 75.4%). The majority of eligible foreign migrant workers, who were not included, were absent mainly either because they were staying in Vietnam at the time of the visit or because they appeared to be no longer living in the study districts. Of the participants, only one participant who withdrew from an interview survey was excluded. Thus, the present study analyzed the data of 391 participants.

### Data collection

A community-based survey was performed between February and June, 2015. The survey included a questionnaire-based interview with a blood examination of each study participant. The questionnaire was first developed in English and then was translated into Lao, Vietnamese, and Chinese languages. Whenever a participant was able to communicate in Lao language, Lao version questionnaire was used. Healthcare professionals including two fluent Vietnamese speakers, who belong to a Lao governmental institution, worked as surveyors. Prior to data collection, the surveyors were trained for a day on data collection procedures. The questionnaire included questions on migration characteristics, socio-demographic and economic characteristics, behavior for malaria prevention and treatment, general healthcare-seeking behavior, and forest activity. Most of the questions were presented with response options: For example, the response options for most of the questions on preventive behavior were “Everyday,” “Often,” “Sometimes,” and “Never.” Questions pertaining to general healthcare-seeking behavior and forest activity were added to the questionnaire during the data collection stage, meaning that these questions were not asked of participants (*n* = 77) in the first study district, i.e., Thapangthong district. The reason for adding the questions was that the research team realized the need for collecting information more comprehensively, as no participant was found to be positive by rapid diagnostic test and a few participants reported a malaria-like illness episode in the first district. The blood examination included a rapid diagnostic test (BinaxNOW® Malaria test) and PCR assay. The PCR assays were performed as described elsewhere [11]. Rapid diagnostic tests were used to provide on-site diagnosis, whereas PCR assays were used to determine malaria infection as the gold standard: The limit of detection for rapid diagnostic tests is generally in the order of 100 parasites/μl, whereas lab-based PCR methods generally have limits of detection of < 5 parasites/μl [12].

### Statistical analysis

Bivariate and multivariate analyses were carried out to examine factors associated with each participant’s self-reported malaria-like illness episode that occurred in the Lao PDR. Factors examined in the analyses were age (< 29 years, 30–39 years, 40–49 years, or > 50 years), gender (male or female), occupation (factory worker, trader/shopkeeper, plantation worker, farmer, construction worker, or wood cutter/forest worker), use of bed net (every day, often, sometimes, or never), and experience of forest activity (yes or no). The odds ratio (OR) and 95% confidence interval (CI) of the outcome (malaria-like illness episode) for each factor were estimated using a mixed-effect logistic regression analysis, with the district-level clustering being treated as a random effect. For the multivariate analysis, all of the factors were entered into the model. These analyses were performed using the STATA MP 12 software program (StataCorp LP, College Station, TX, USA).

## Results

### Blood examination

No participants tested positive for malaria either by rapid diagnostic test or by PCR assay. Therefore, the positive rate of malaria infection was 0% (0/391).

### General characteristics

Most of the participants (75.7%) were men, with a median age of 31 years old (Table 1), and most (92.6%) were Vietnamese, with the remaining participants being either Chinese or Thai. The most common occupation was sawmill factory worker (24.6%), followed by trader/shopkeeper (21.5%), industrial factory worker (18.4%), plantation worker/farmer (16.4%), and repairer (5.1%). The location of the participants included both urban (i.e., township) and rural areas (i.e., forest fringe).

**Table 1:** General characteristics of study participants

Characteristics	Number (*n* = 391)	Percentage
Gender		
Male	296	75.7
Female	95	24.3
Age (years)		
Median (interquartile range)	31 (25 to 41)	
Nationality		
Vietnamese	362	92.6
Chinese	23	5.9
Thai	6	1.5
Occupation		
Sawmill factory worker	96	24.6
Trader/shopkeeper	84	21.5
Industrial factory worker	72	18.4
Plantation worker/farmer	64	16.4
Repairer	20	5.1
Furniture factory worker	18	4.6
Construction worker	11	2.8
Wood cutter	8	2.0
Other	18	4.6
Study site		
Thapangthong district	77	19.7
Xepon district	104	26.6
Nong district	108	27.6
Phin district	102	26.1

### Migration characteristics

When migrating to the study district, most of the participants were accompanied either by a friend (36.1%) or by a family member (33.2%) (Table 2). At the time of the survey, the participants’ median length of stay in the study district was 405 days (interquartile range, 183 to 1207 days). The majority of the participants (85.9%) had not worked in a province other than the study province, nor had the majority (92.6%) worked in a foreign country other than the Lao PDR.

**Table 2:** Migration characteristics of the study participants

Characteristics and behavior	Number (*n* = 391)	Percentage
Migration to the study site		
With friend	141	36.1
With family member	130	33.2
Alone	118	30.2
With company staff	2	0.5
Length of stay in the study site in days, median (interquartile range)	405 (183 to 1207)	
Experience of working in a province other than the study province		
Yes	55^a^	14.1
No	336	85.9
Unknown	1	0.3
Experience of working in a foreign country other than Lao PDR		
Yes	28^b^	7.2
No	362	92.6
Unknown	1	0.3

^a^The provinces were Salavan, Champasack, Vientiane City, Attapeau, Bolikhamxay, Khammuan, and others

^b^The foreign countries were Thailand, Cambodia, Malaysia, and others

### Malaria preventive behavior

Although most of the participants (62.7%) used a bed net daily, non-users (10.2%) and infrequent users (7.2%) also existed (Table 3). The use of mosquito repellent skin lotion was uncommon: 86.7% never used such lotions. Eleven participants (2.8%) had taken an anti-malaria medicine for malaria prevention when living in the Lao PDR: three participants reportedly took quinine, and the remainder did not remember the name of the medicine. These 11 participants did not take the medicine frequently.

**Table 3:** Malaria preventive behavior of the study participants

Characteristics	Number (*n* = 391)	Percentage
Frequency of bed net use		
Everyday	245	62.7
Often	78	19.9
Sometimes	28	7.2
Never	40	10.2
Frequency of mosquito repellent skin lotion use		
Everyday	4	1.0
Often	10	2.6
Sometimes	31	7.9
Never	339	86.7
Unknown	7	1.8
Experience of taking anti-malaria medicine for malaria prevention in Lao PDR		
Yes	11^a^	2.8
No	375	95.9
Unknown	5	1.3
Frequency of taking the anti-malaria medicine (*n* = 11)		
Everyday	0	0.0
Often	0	0.0
Sometimes	4	36.4
Rarely	6	54.5
Unknown	1	9.1

^a^Three people took quinine as the medicine, whereas the remaining 8 people did not remember the name of the medicine

### Characteristics of bed nets

Of the 345 bed nets that the participants used, most (*n* = 253, 73.3%) were conventional non-treated nets (data not shown in tables). Only 49 (14.2%) of the participants used an insecticide-treated net. The insecticide-treated status was unclear for the 43 (12.5%) remaining bed nets. Over half of the bed nets used were obtained in Vietnam (*n* = 195, 56.5%), whereas fewer bed nets were obtained in the Lao PDR (*n* = 25, 7.2%) and Thailand (*n* = 23, 6.7%). The place of origin of 102 (29.6%) bed nets was unclear.

### Treatment-seeking behavior for malaria-like illness

Thirty-nine participants (10.0%) reported a malaria-like illness episode that occurred while living in the Lao PDR (Table 4). The most common measure taken for the episode was to visit a hospital/health center in the Lao PDR (*n* = 23, 59.0%), followed by conducting self-medication alone (*n* = 6, 15.4%) and taking a rest alone (*n* = 6, 15.4%). Among the 24 participants who visited a hospital/health center, nearly half (*n* = 11, 45.8%) conducted self-medication prior to the visit.

**Table 4:** Treatment-seeking behavior of the study participants for a self-reported malaria-like illness episode

Characteristics	Number (*n* = 391)	Percentage
Experience of suffering from malaria-like illness in their home countries		
Yes	18	4.6
No	370	94.6
Unknown	3	0.8
Experience of suffering from malaria-like illness in Lao PDR		
Yes	39	10.0
No	352	90.0
Behavior of the study participants for the malaria-like illness episode that occurred in Lao PDR (*n* = 39)		
Visited a health center/hospital in Lao PDR	23	59.0
Visited a health center/hospital in Vietnam	1	2.6
Conducted self-medication alone	6	15.4
Took a rest alone	6	15.4
Unknown	3	7.7
Conducted self-medication prior to the visit to the health center/hospital in Lao PDR/Vietnam (*n* = 24)		
Yes	11	45.8
No	13	54.2

The majority of the participants (*n* = 28, 71.8%) who reported a malaria-like illness episode had not worked in a province other than the study province (data not shown in tables).

### General healthcare-seeking behavior

Of the 314 participants from the Xepon, Nong, and Phin districts, the majority (*n* = 279, 88.9%) reported that they had not experienced seeking healthcare service from Lao medical staff (Table 5). Among the participants who had sought healthcare, the major healthcare service they received was treatment (*n* = 24, 80.0%). In response to the hypothetical question “If you get sick, then would you like to utilize a Lao hospital/health center?”, 61.8% of the participants answered “Yes,” whereas 18.2% answered “No” and 18.5% answered “Not sure.”

**Table 5:** General healthcare-seeking behavior of the study participants

Characteristics	Number (*n* = 314)	Percentage
Experience of seeking healthcare service from Lao medical staff		
Yes	30	9.6
No	279	88.9
Unknown	5	1.6
Healthcare service he/she received (*n* = 30)		
Medical check-up	1	3.3
Treatment	24	80.0
Consultation	0	0.0
Obtained drug	2	6.7
Unknown	3	10.0
If you get sick, would you like to utilize a Lao health center/hospital?		
Yes	194	61.8
No	57	18.2
Not sure	58	18.5
Unknown	5	1.6

### Forest activity

Of the 314 participants from Xepon, Nong, and Phin districts, 41 (13.1%) reported an experience of working in the forest while living in the Lao PDR (data not shown in tables). The objectives of the forest work were wood cutting (*n* = 12, 29.3%), plantation building (*n* = 11, 26.8%), swidden (*n* = 9, 22.0%), seeking forest products (*n* = 5, 12.2%), and others (*n* = 4, 9.8%).

### Factors associated with malaria-like illness episodes

Bivariate analysis showed a statistically significant association between malaria-like illness episodes and age, occupation, and forest activity in the Lao PDR (Table 6). The participants aged 50 years or older were significantly more likely to have had a malaria-like illness episode compared to those aged 29 years or younger (OR 6.46, 95% CI 2.19 to 19.05). Additionally, construction workers/wood cutters were significantly more likely to have had a malaria-like illness episode compared to factory workers (OR 5.96, 95% CI 1.08 to 32.79). The participants who were involved in forest-related activities while living in the Lao PDR were significantly more likely to have had a malaria-like illness episode compared to those who were not involved in such activities (OR 5.53, 95% CI 2.31 to 13.25).

**Table 6:** Factors associated with malaria episodes that occurred in Lao PDR among the study participants (*n* = 308)

Variables		Bivariate analysis		Multivariate analysis	
	Malaria episode (%)	Odds ratio	95% CI^a^	Odds ratio	95% CI^a^
Gender					
Female	5.6	1.00	Reference	1.00	Reference
Male	11.9	2.23	0.75–6.64	1.53	0.42–5.61
Age (years)					
< 29	5.6	1.00	Reference	1.00	Reference
30–39	9.9	1.82	0.65–5.09	1.32	0.44–3.96
40–49	13.8	2.74	0.97–7.72	2.01	0.62–6.46
> 50	29.0	6.46	2.19–19.05	4.90	1.54–15.54
Occupation					
Factory worker	7.1	1.00	Reference	1.00	Reference
Trader/shopkeeper	8.5	1.09	0.23–5.19	2.06	0.50–8.46
Plantation worker/farmer	17.2	2.54	0.88–7.31	1.29	0.44–3.83
Construction worker/wood cutter	33.3	5.96	1.08–32.79	4.81	0.88–26.30
Other	11.5	1.56	0.32–7.54	1.39	0.29–6.60
Use of bed net					
Every day/often	11.1	1.00	Reference	1.00	Reference
Sometimes/never	9.6	0.74	0.32–1.74	0.76	0.30–1.92
Forest activity in Lao PDR					
No	6.7	1.00	Reference	1.00	Reference
Yes	26.8	5.53	2.31–13.25	5.24	1.91–14.34

^a^Confidence interval

Multivariate analysis confirmed the significant association of malaria-like illness episodes with age (OR 4.90, 95% CI 1.54 to 15.54) and forest activity (OR 5.24, 95% CI 1.91 to 14.34), whereas the association between malaria-like illness episodes and occupation (construction workers/wood cutters) became insignificant in the multivariate analysis (OR 4.81, 95% CI 0.88 to 26.30).

## Discussion

In the present study involving 391 foreign migrant workers from Thapangthong, Xepon, Nong, and Phin districts in 2015, no participants tested positive for malaria, even though a number of studies that were conducted with the general Laotian population in these districts between 2013 and 2015 reported substantial positive rates of *Plasmodium* infections. One study that was conducted with 891 villagers in 10 villages in Xepon district reported a positive rate of 6% [13], and a study conducted with 888 villagers in 18 villages in Thapangthong and Nong reported a positive rate of 20% [14]. Therefore, the study participants of the present study can be considered to be at low risk of malaria infection and registered foreign migrant workers in the study province might not be a high-risk group.

None of the participants in the present study were infected with malaria at the time of blood examination. A possible explanation for the absence of positive participants is that the present study did not include unregistered/illegal foreign migrant workers, of whom there are estimated to be 24,000 among the 54,000 foreign migrants in the Lao PDR [6]. These workers are more likely to have malaria infection as compared to registered/legal foreign migrant workers because they deliberately avoid contact with Lao governmental facilities. Another possible reason is that the majority of the study participants were not involved in logging, dam construction, or mining, which are thought to be high-risk occupations [9].

A further study is necessary that targets unregistered/illegal foreign migrant workers in the Lao PDR. Sampling will be a challenge, however, because these workers are hard to reach. A study conducted in Thailand to explore knowledge, perception, and practices pertaining to malaria [15] included both registered and unregistered foreign migrant workers. That study used respondent-driven sampling, which is an extension of snowball sampling. It may be possible to gain access to unregistered/illegal foreign migrant workers in the Lao PDR if snowball sampling is applied.

Among the study participants, 62.7% used a bed net daily. This percentage is lower than those reported from studies conducted with the general Laotian population in the same districts: 82.7% in villages with high incidence and 82.3% in villages with low incidence of malaria in Xepon [16] and 76.8% in villages of Nong and Thapangthong districts [14].

In the present study, 10% of the participants reported a malaria-like illness episode that had occurred in the Lao PDR. Because 86% of the participants had no experience of working in a province other than the study province, they may have contracted malaria-like illness in the study province. Some of these malaria-like illness episodes could be due to malaria because such illness episodes were significantly associated with the involvement in forest-related activities. In the study province, involvement in forest-related activities is known to be a risk factor of malaria [14, 16].

Approximately 95% of the study participants had reportedly never experienced malaria in their mother countries. Thus, they may not have been aware of the risk of malaria while living in the Lao PDR. Foreign migrant workers who live in malaria-endemic districts in the Lao PDR should be educated about the risk of contracting malaria when working in the forest in the Lao PDR.

Approximately 60% of the participants used a Lao healthcare facility such as a health center or hospital for treatment of a self-reported malaria episode. This percentage could be reasonable because more than half of the participants had been living in the Lao PDR for over a year and thus might not have had a critical language barrier, which is known to be a major barrier for migrants for the use of the healthcare services of a host country [17–19]. Another possible reason is that the use of a public healthcare facility for malaria treatment is common among some Vietnamese. A study conducted with the general Vietnamese population in Quang Tri province, which borders the study province, showed that 90% of respondents used a public healthcare facility for treatment of a malaria episode [20].

The present study has some limitations. First, because the survey period did not coincide with the high transmission season (often between June and August) [21], the present study could underestimate the prevalence of malaria infection. Second, the participation rate was 75.4%, suggesting that there is a possibility of selection bias. Because only limited information was available on the eligible workers who were not included, the present study was unable to estimate the impacts of this bias on the study findings. However, the impacts might not be large, as the major reason for the non-participation was not the refusal but the absence. Additionally, the survey widely covered the location of participants. Finally, apart from blood examinations, the data collection relied on the self-report of participants. Therefore, some malaria-like illness episodes might not be due to malaria and the information on behavioral and migration characteristics might not be accurate. Despite these limitations, the present study is, to be the best knowledge, the first study that reported the prevalence of malaria infection, migration characteristics, and preventive and treatment behaviors among foreign migrant workers in Lao PDR.

## Conclusions

The foreign migrant workers who are registered at local police departments are unlikely to play a major role in maintaining local transmissions and spreading drug-resistant malaria in the study province. However, some of them were involved in forest-related activities that were significantly associated with malaria-like illness episodes, suggesting that they are potentially at risk of malaria. For treatment of these episodes, the majority of the study participants used a local Lao healthcare facility. The use of a bed net was common, although their nets were mostly conventional untreated ones. Because 94.6% of the participants had not experienced malaria in their mother countries, they may not be very aware of the risk of malaria while living in the Lao PDR. Therefore, the Lao National Malaria Control Program should educate foreign migrant workers about the risk of acquiring malaria in the Lao PDR. Because the present study included only registered foreign migrant workers, a further study that targets unregistered foreign migrant workers is also warranted.
